# HC-1119, a deuterated Enzalutamide, inhibits Migration, Invasion and Metastasis of the AR-positive triple-negative breast Cancer cells

**DOI:** 10.1007/s11033-022-07749-8

**Published:** 2022-08-12

**Authors:** Xuehong Wu, Wanru Feng, Mao Yang, Xunxi Liu, Mengdi Gao, Xinghai Li, Lin Gan, Tao He

**Affiliations:** 1 Institute for Cancer Medicine, School of Basic Medical Sciences, Southwest Medical University, 646000 Luzhou, Sichuan China; 2 Department of Biochemistry and Molecular Biology, School of Basic Medical Sciences, Southwest Medical University, 646000 Luzhou, Sichuan China; 3 Hinova Pharmaceuticals Inc, No. 2-3, 4th Floor, Rongyao Tower 1, 5 South Keyuan Road, 610041 Chengdu, China; 4 Mindong Hospital Affiliated to Fujian Medical University, 355000 Fuan, Fujian Province China

**Keywords:** Triple-negative breast cancer (TNBC), Androgen receptor (AR), HC-1119, Deuterated enzalutamide, Metastasis

## Abstract

Triple-negative breast cancers (TNBCs) are aggressive, and they develop metastasis at earlier stages, relapse more frequently, and exhibits poorer prognosis than other subtypes of breast cancer. Due to the lack of estrogen receptor for endocrine therapy and HER2 for targeted therapy, new targeted therapies for TNBCs are urgently needed. Enzalutamide is a second-generation androgen receptor (AR) inhibitor, and HC-1119 is a new synthetic deuterated enzalutamide. Owing to the isotope effect, HC-1119 has many advantages over enzalutamide, including slow metabolism, high plasma concentration and low brain exposure. However, the efficacy of HC-1119 in inhibition of AR function in triple-negative breast cancer (TNBC) has not been studied. In this study, we found high-level AR expression in both Hs578T and SUM159PT TNBC cell lines. Activation of AR by dihydrotestosterone (DHT) in both cell lines increased AR protein, induced AR-nuclear localization, enhanced cell migration and invasion in culture, and promoted liver metastasis in mice. Importantly, cotreatment with HC-1119 of these cells efficiently abolished all of these effects of DHT on both Hs578T and SUM159PT cells. These results indicate that HC-1119 is a very effective new second-generation AR antagonist that can inhibit the migration, invasion and metastasis of the AR-positive TNBC cells.

**Supplementary Information:**

The online version contains supplementary material available at 10.1007/s11033-022-07749-8.

## Introduction

According to the International Agency for Research on Cancer (IARC), breast cancer was the most commonly diagnosed cancer and the fifth leading cause of cancer death worldwide in 2020 [1]. There are three major types of breast cancer: hormone receptor positive (HR+) breast cancer that expresses estrogen receptor (ER) with or without progesterone receptor (PR), human epithelial growth factor receptor 2 positive (HER2+) breast cancer, and triple-negative ( lack of estrogen receptor-α (ERα), progesterone receptor (PR), and human epidermal growth factor receptor-2 (HER2) amplification) breast cancer (TNBC). TNBC accounts for about 10–15% of all breast cancers and is the most malignant subtype [2, 3]. Because of the lack of ER and PR expression as well as HER2 amplification, both the endocrine and anti-HER2 therapies are inapplicable to the treatment of TNBC [4, 5]. Therefore, new therapeutic strategy was eagerly needed for improving the treatment of TNBC.

Androgen receptor (AR) signaling regulates multiple bioprocesses such as the development and maintenance of male sexual organs, bones, and reproductive function, and also helps ovarian follicles in female to develop and ovulate normally [6, 7]. AR is an androgen-inducible transcription factor that directly binds to DNA and regulates many gene transcriptions [8, 9]. Abnormal AR signaling has been reported in cancer development. For example, the TMPRSS2 gene is a target gene of AR. Therefore, in prostate cancers with the TMPRSS2-ERG fusion oncogene, activation of AR signaling upregulates the expression of this fusion oncoprotein, leading to enhanced proliferation and survival and prostate cancer cells [10][11]. Interestingly, recent studies reported that AR is expressed in 70–90% of all breast cancers [12–15] and 22–35% of TNBC [16, 17], suggesting that AR could be a potential therapeutic target for treating breast cancer [18, 19]. Indeed, a full clinical response has been achieved after a patient with metastatic AR-positive TNBC was treated for 4 months with bicalutamide, a first-generation nonsteroidal AR inhibitor [20], and a 19% clinical benefit rate (CBR) has been observed after 424 patients with ER/PR-negative breast cancers were treated with bicalutamide for 6 months [21]. Further, treatment with enzalutamide, a second-generation AR inhibitor, has increased CBR to 33% and improved progression-free survival (PFS) and overall survival (OS) of patients with AR-positive TNBCs [22].

Enzalutamide is a 2nd generation AR inhibitor approved and extensively for treating metastatic castration-sensitive prostate cancer (mCSPC), non-metastatic castration-resistant prostate cancer (nmCRPC), and metastatic castration-resistant prostate cancer (mCRPC) [23, 24], HC-1119 is a synthetic molecule of deuterated enzalutamide that is currently used in a Phase III clinical trial for mCRPC treatment [NCT03850795]. Owing to the kinetic isotope effect, HC-1119 has a significantly slower metabolism in cells, and a markedly elevated plasma concentration and a decreased brain exposure after administrated in vivo [25]. Because of these unique pharmacokinetic features, HC-1119 can be given to patient at a significantly reduced dosage with reduced risks. In the Phase III trial, HC-1119 was given at a dose of 80 mg per day, which was much lower than the regular dose of 160 mg enzalutamide per day used to treat prostate cancer patients. In Phase I trial, the reduced brain exposure of HC-1119 versus the regular enzalutamide is associated with a lower risk to induce epilepsy and other central nervous system (CNS)-related side effects [25].

In this study, we performed both in vitro and in vivo experiments to evaluate how HC-1119 can effectively inhibit the growth, migration, invasion, and metastasis of AR-positive TNBC cells. We found that HC-1119 can significantly decrease the migration and invasion capabilities of the AR-positive TNBC cells by impeding the epithelial-mesenchymal transition (EMT) phenotype and inhibit the metastasis of these TNBC cell-derived tumors in mice. Our results demonstrate that HC-1119 may be useful for treating AR-positive TNBC.

## Materials and methods

### Cell culture

SUM159PT, Hs578T, T47D, MCF7, MDA-MB-231, MDA-MB-468, MDA-MB-453 and 22Rv1 cell lines were obtained from Chinese Academy of Medical Sciences. SUM159PT, Hs578T, T47D, MDA-MB-231, MDA-MB-468 and MDA-MB-453 cells were cultured in DMEM (SH30022.11, Hyclone) with 10% fetal bovine serum (FBS) (11,041 − 8611, EVERY GREEN). 22Rv1 cells were cultured in RPMI medium 1640 containing 10% FBS, 1% glutamax and 1% sodium pyruvate. MCF7 cells were cultured in MEM medium with 10% FBS.

### Immunoblotting

Cells were lysed with RIPA buffer (25 mM Tris HCl (pH 7.6), 1% sodium deoxycholate, 150 mM NaCl and 0.1% SDS) containing protease inhibitor cocktail (Roche, Basel, Switzerland). Total cell lysates with 60–80 µg protein were boiled for five minutes in SDS-PAGE reducing buffer, and loaded to SDS-PAGE gels. Separated proteins in the gel were transferred onto a nitrocellulose membrane. Primary antibodies used for immunoblotting were against androgen receptor (#3125, Cell Signaling Technology), vimentin (#5741, Cell Signaling Technology), fibronectin (15613-1-AP, Proteintech), Keratin 8/18 (#4546, Cell Signaling Technology), and GAPDH (#2118S, Cell Signaling Technology). Fluorescence-labeled secondary anti-mouse IgG antibody (LI-COR Biosciences, Lincoln, NE, USA) was used to detect the primary antibodies. The fluorescence intensity of the secondary antibody was assayed using an Odyssey Imaging System (LI-COR).

### RT-qPCR

Total RNA was extracted by Trizol reagent (Invitrogen). One g RNA was used in reverse transcription. The relative concentration of a mRNA was measured by real-time quantitative PCR (qPCR) and normalized to GAPDH mRNA. The 5’ primer sequences, 3’ primer sequences, and the fluorescence-labeled probes were 5’-gccttgctctctagcctcaa, 5’-ggtcgtccacgtgtaagttg, and probe 14# (04688970001, Roche) for AR, and 5’-agccacatcgctcagacac, 5’-gcccaatacgaccaaatcc, and probe 60# for GAPDH.

### Transwell assay

Prior to drug treatment, all cells were cultured in phenol red-free DMEM medium (319-051-CL, Multicell) containing 10% charcoal-stripped FBS (04-201-1B, CSS, Biological Industries) for 48 h to exhaust androgen. Dihydrotestosterone (DHT) and HC-1119 were dissolved in DMSO. Hs578T cells were pretreated with 5 nM DHT and/or 0.5 µM HC-1119 or vehicle (DMSO) for 24 h. SUM159PT cells were pretreated with 5 nM DHT and/or 10 µM HC-1119 or vehicle (DMSO) for 24 h. For invasion assay, 5 × 104 pretreated cells in 200 µl of phenol red-free DMEM were seeded into the upper chamber with a membrane precoated with 20 µl of PBS-diluted Matrigel (1:1 dilution) (BD Corning, NY, USA). For migration assay, 3 × 104 pretreated cells in the same medium were seeded into the upper chamber without Metrigel on the bottom membrane. The lower chamber was filled with phenol red-free DMEM containing 10% charcoal-stripped FBS (CSS). After cultured in a 37 °C incubator with 5% CO2 for 24 h, cells on the upper surface of the membrane were removed with a cotton swab. Cells on the lower surface of the membrane were fixed in 500 µl methylal, washed in 1 ml PBS, dried, and stained in crystal violet solution (crystal violet: PBS = 1:10) for 3 h. The stained cells were imaged under a microscope and quantitatively analyzed.

### Immunofluorescence staining of cells

Cells (4 × 104) were seeded on glass coverslips in a 24-well culture plates, treated with DHT and/or HC-1119 for one hour. The treated cells were washed with TBST, fixed in 4% formaldehyde solution and permeabilized with 0.3% Triton X-100 in TBST, and blocked with 5% BSA in TBST for 1.5 h. Cells on coverslips were incubated overnight with an androgen receptor antibody (#5153S, Cell Signaling Technology), followed by incubation with a goat anti-Rabbit IgG (H + L) secondary antibody (UD285216, Invitrogen) for 1 h, and DAPI staining. Finally, the stained cells on coverslips were examined with a confocal fluorescence microscope (Leica Microsystems, Germany).

### Xenograft tumor growth

Five- to six-week-old female BALB/c nude mice were purchased from Vital River Laboratory Animal Technology, Beijing, China. One week later, 2 × 106 SUM159PT cells were injected into one of the forth pair mammary gland fat pads of each mouse. When the volume of the tumors derived from these injected cells reached 100 mm3, mice in group 1 were treated with DHT (7 mg/kg, i.p., once every other day) or equal volume of vehicle (dosing in 5% Dimethyl sulfoxide (DMSO), 30% Poly- ethylene glycol 400(PEG400), 5% Tween80 and 60% ddH2O). Mice in group 2 were treated with HC-1119 (50 mg/kg/day, oral feeding) or equal volume of vehicle (containing 5% DMSO, 18% PEG400, 1% Tween80, 60% of 0.5% Carboxymethylcellulose (CMC) sodium, 15% of 20% Vitamin E-TPGS in 20 mM citrate buffer (pH 4.0), 1% Polyvinylpyrrolidone (PVP)). Mice in group 3 were treated with both DHT and HC-1119 in the same doses given to mice in groups 1 and 2. Tumor length and width were measured 3 times a week by using a caliper. Tumor volume was calculated using the formulae: volume = 1/2×(length×width2). Mice were euthanized by carbon dioxide asphyxiation, and the tumors, lungs and livers were collected for histopathological examination and biochemical analysis.

### Hemotoxylin and eosin (H&E) staining and immunohistochemistry

The harvested xenograft tumors and other tissues were fixed in 4% paraformaldehyde, embedded in paraffin, and cut into 5 μm-thick sections. Sections on glass slides were deparaffinized using xylene and rehydrated in a series of ethanol dilutions and water. Some xenograft tumor and liver sections were stained with H&E for histopathological examination. Other tumor sections were soaked in 10 mM sodium citrate (pH = 6.0) and heated in a high-pressure cooker for 4 min for antigen retrieval, and treated with 3% H2O2. After blocked with 5% bovine serum albumin (BSA) for 1 h, sections were incubated overnight with AR antibody (#3152, Cell Signaling Technology) at 1:200 dilution in TBS containing 5% BSA at 4 ℃. After washed and incubated with the biotinylated goat-anti-rabbit IgG secondary antibody, the immunostaining signal was visualized by using a DAB kit (#11724S, Cell Signaling Technology). The sections were further counterstained with Hematoxylin, dehydrated in a series of ethanol solutions and ethanol, and mounted with Permount for microscopy and imaging.

### Statistical data analysis

Every experiment was repeated at least three times. Data analysis was conducted using GraphPad Prism Version 6.0. Student’s t-test and One-Way ANOWA were used to compare the differences between two and more groups, respectively. Statistical significance was accepted if P < 0.05.

## Results

### DHT treatment increases AR protein expression in TNBC cells

**Fig. 1:**
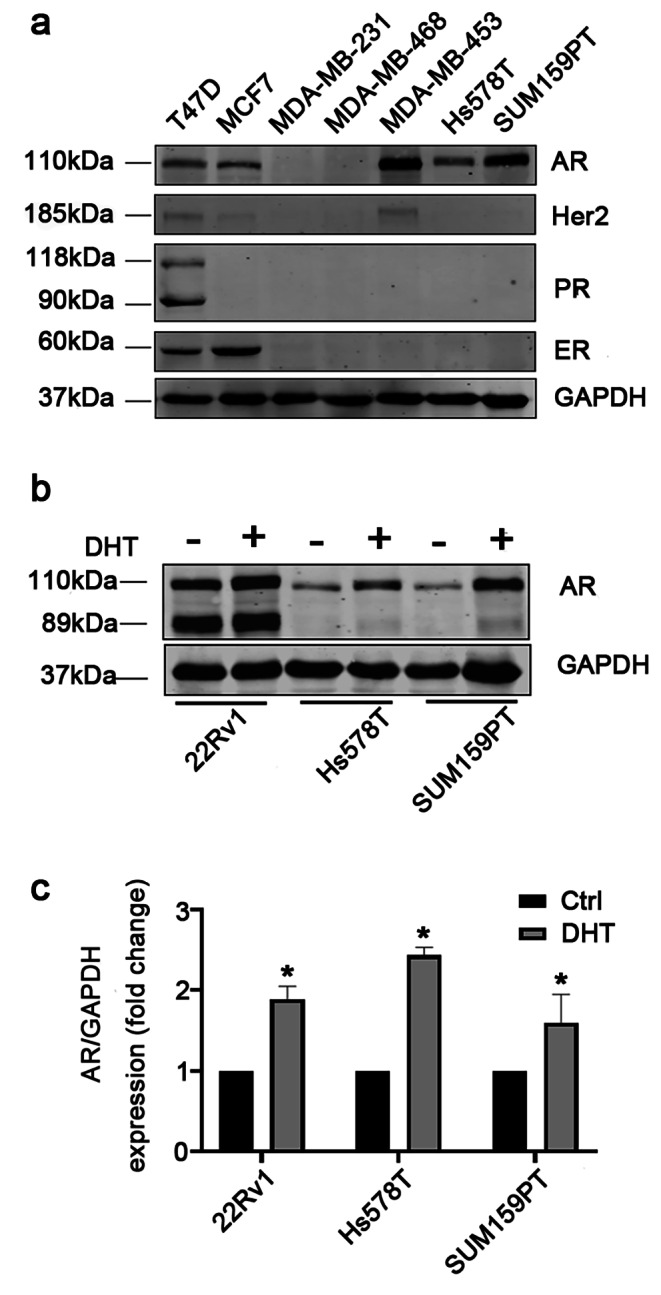
**Examination of AR expression in different breast cancer cell lines (a)** Western blot assays of AR, HER2, PR and ER in the indicated cell lines. Cells were cultured in media with 10% FBS. Whole cell lysates were analyzed by western blot. GAPDH was used as a loading control. **(b)** DHT treatment increased AR protein in the indicated cell lines. The 22Rv1 prostate cancer cell line served as a positive control. Cells were cultured in phenol red-free medium containing 10% charcoal-stripped FBS (CSS) for 48 h and then treated with vehicle or 5 nM of DHT for 24 h. Whole cell lysates were subjected to Western blot assay using AR antibody. **(c)** The average ratios of the AR band intensity to the GAPDH band intensity, which were calculated from the data of three independent assays. *, p < 0.05 by unpaired Student’s t-test

In order to choose appropriate breast cancer cell lines for this study, we examined ER, PR, HER2 and AR expression in multiple breast cancer cell lines. Among the TNBC cell lines, MDA-MB-231 and MDA-MB-468 cells do not express AR, while Hs578T and SUM159PT cells express a moderate-level and a high-level AR protein, respectively (Fig. 1 A). Similar to that observed in the 22Rv1 prostate cancer cells, treatment with 5 nM of DHT, a natural ligand of AR, significantly increased the expression levels of AR protein in Hs578T and SUM159PT TNBC cells (Fig. 1 B and C). These results demonstrate that androgen stimulation can significantly increase AR expression in these cells and Hs578T and SUM159PT cell lines are appropriate for studying the role of AR antagonist in TNBC cells.

### HC-1119 treatment abolishes the DHT-induced AR expression and nuclear translocation in AR-positive TNBC cells

**Fig. 2:**
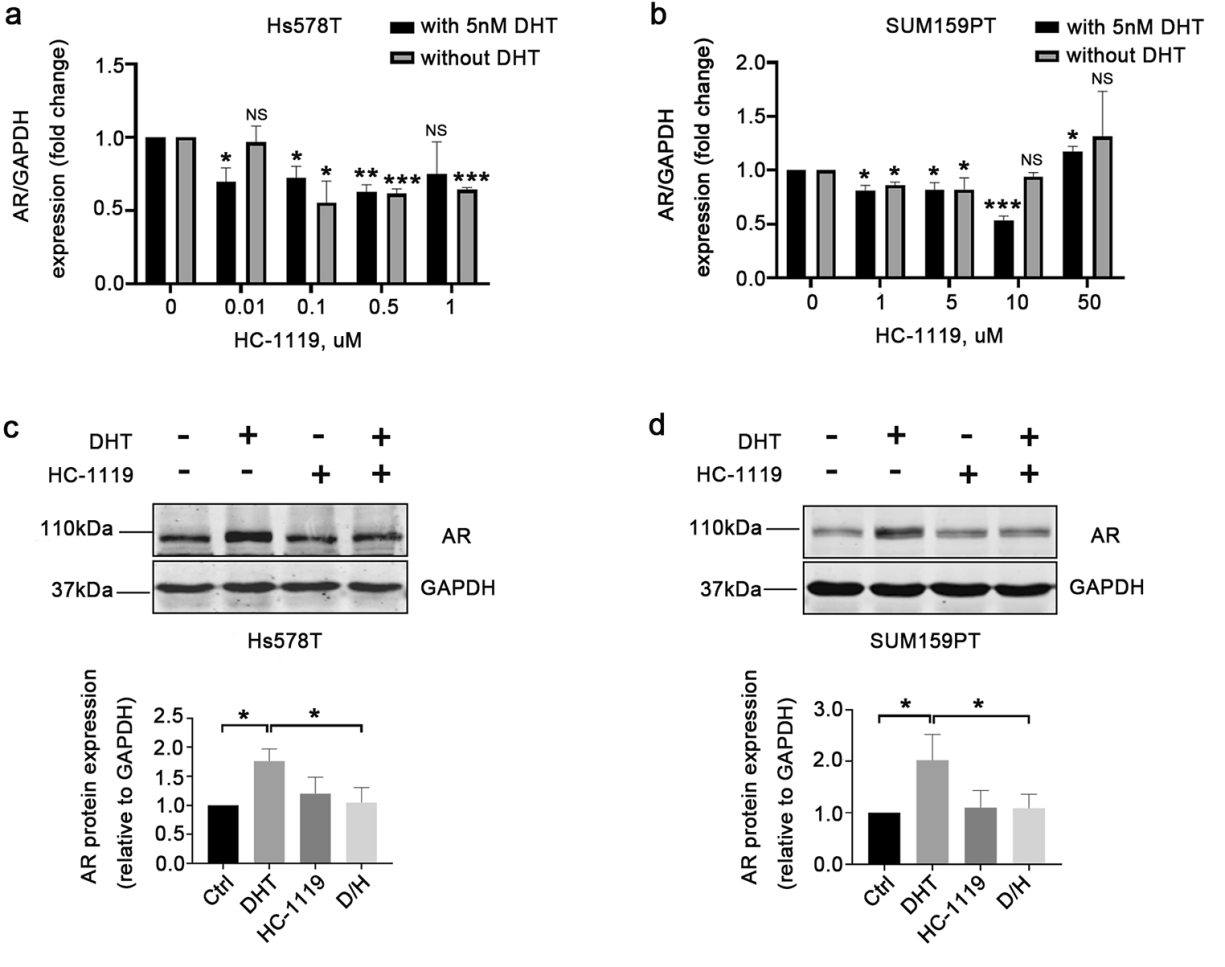
**HC-1119 treatment inhibited the increase in AR protein elevated by DHT treatment in AR-positive TNBC cells** Hs578T **(a)** and SUM159PT **(b)** cells were cultured in a medium with 10% CSS for 48 h, and then treated with vehicle, 5 nM of DHT, 0.5 μm (for Hs578T cells) or 10 μm (for SUM159PT cells) of HC-1119, or both DHT and HC-1119 as indicated for 24 h. Whole cell lysates were prepared from the treated cells and analyzed by Western blot using AR antibody. GAPDH served as a loading control. Experiments were repeated at least three times and the representative bands of Western blot assays are presented. *, p < 0.05 by One-Way ANOVA

To test the antagonistic effect of HC-1119 on DHT, we treated both Hs578T and SUM159PT TNBC cells with vehicle, DHT and/or HC-1119 and examined the expression levels of AR protein in these treated cells. Again, DHT treatment increased AR protein. However, HC-1119 treatment alone did not alter the levels of AR protein in these cells. Importantly, HC-1119 treatment abolished the DHT-induced AR expression in these cells (Fig. 2 A and B). These results indicate that HC-1119 can effectively inhibit DHT-induced increase in AR protein expression in AR-positive TNBC cells.

**Fig. 3:**
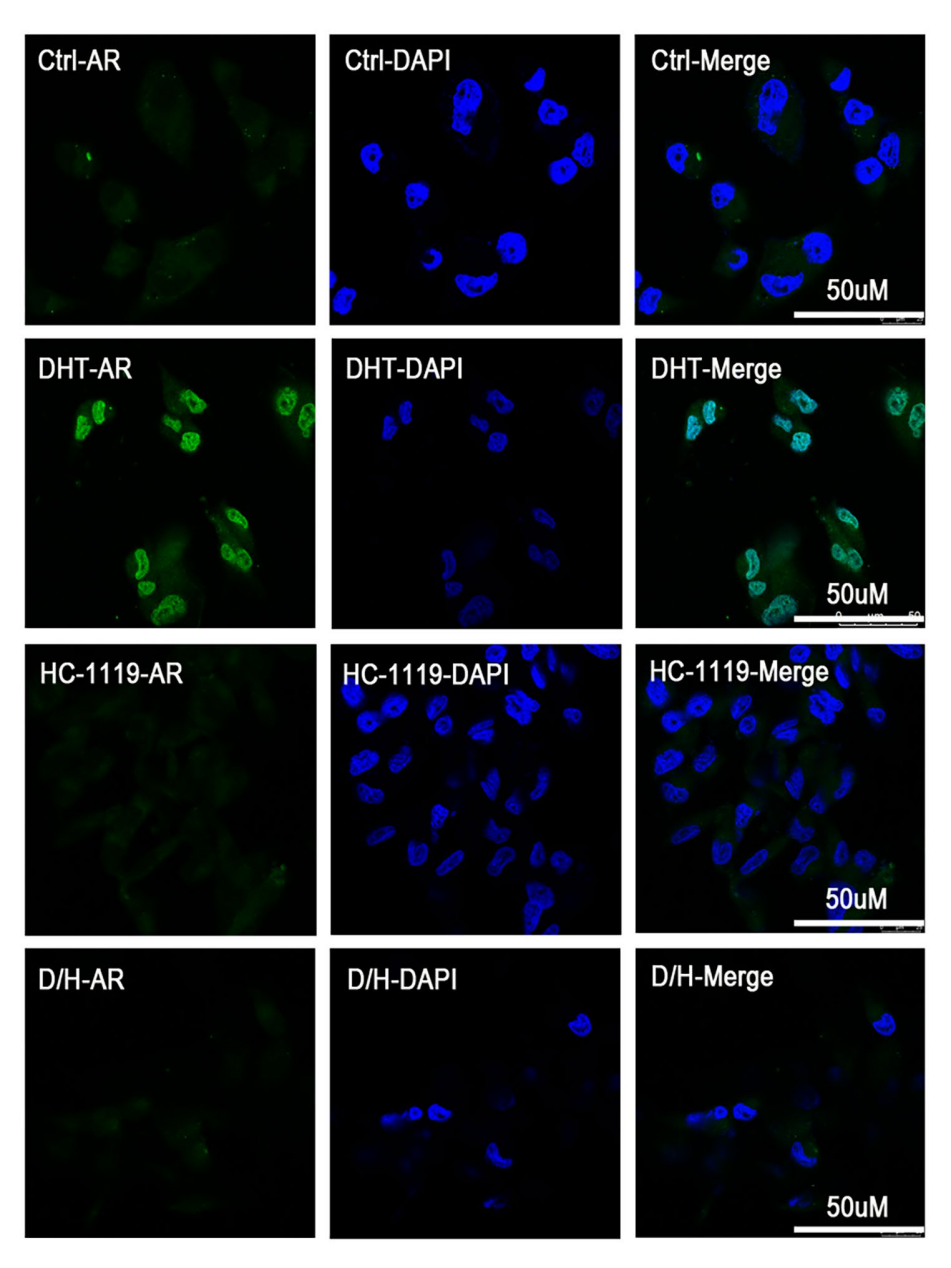
**HC-1119 treatment suppresses DHT-induced nuclear localization of AR in AR-positive TNBC cells** Hs578T cells were cultured in a medium with 10% CSS for 48 h, and then seeded onto glass coverslips in a 24-well plate, and treated for one hour with vehicle (Ctrl), DHT (5nM), HC-1119 (0.5 μm), or both DHT and HC-1119 as indicated. Treated cells were processed for immunecytofluorescence staining using AR antibody. Images were recorded with a confocal microscope

The translocation of AR from cytoplasm to nucleus is an essential step for the expression of AR-regulated genes, and block of AR nuclear-translocation is a key feature of the second-generation antiandrogens distinct from the first-generation antiandrogens [28]. To determine whether HC-1119 can efficiently inhibit AR nuclear translocation, we treated Hs578T cells with vehicle, DHT, HC-1119, or both DHT and HC-1119 and examined AR subcellular location by performing immunocytofluorescence. In vehicle-treated cells, AR was mainly detected in the cell cytoplasm. In DHT-treated cells, AR was mainly observed in the cell nucleus. In the HC-1119-treated cells, AR was mostly located in the cytoplasm, showing similar distribution as that in vehicle-treated cells. In the DHT and HC-1119-cotreated cells, no AR staining was detected in the nucleus (Fig. 3). These results demonstrate that HC-1119 is a potent second-generation antiandrogen that can efficiently block DHT-induced AR translocation into the nuclei of TNBC cells.

### HC-1119 treatment inhibits DHT-induced migration and invasion of AR-positive TNBC cells

**Fig. 4:**
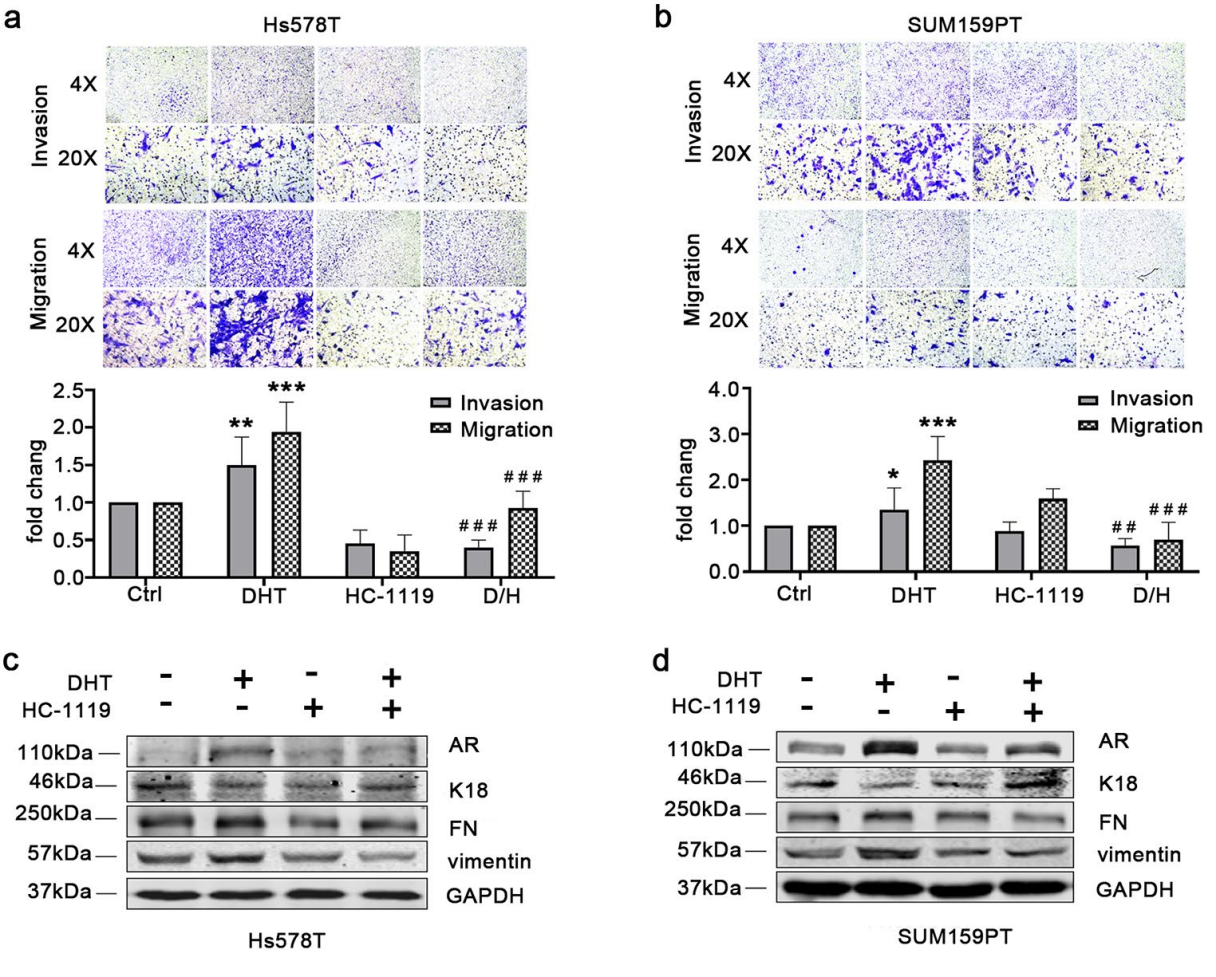
**HC-1119 inhibits DHT-stimulated migration and invasion of AR-positive TNBC cells (a, b)** Effects of HC-1119 on the migration and invasion of AR-positive TNBC cells. Hs578T **(a)** and SUM159PT **(b)** cells in the upper chambers of the transwell plate for migration and invasion assays were treated with vehicle (Ctrl), DHT, HC-1119 or both DHT and HC-1119 as indicated for 24 h. Migrated or invaded cells attached to the lower surface of the membrane were imaged under microscope with 4X and 20X magnifications. The quantitative cell numbers of migrated and invaded cells were normalized to cell numbers of vehicle treated groups. *, **, and ***, p < 0.05, p < 0.01, and p < 0.001, which were compared with Ctrl groups by One-Way ANOVA. ##. and ###, p < 0.01, and p < 0.001, which were compared with DHT groups by One-Way ANOVA. **(c, d)** Hs578T **(c)** and SUM159T **(d)** cells were treated with vehicle (Ctrl), DHT, HC-1119, or both DHT and HC-1119 for 24 h as indicated. Whole cell lysates of the treated cells were analyzed for the indicated proteins by Western blot. Experiments were repeated at least three times and the representative images were presented. Each band intensity on Western blot was quantitatively measured by densitometry, and the average band intensity of each protein was normalized to the band intensity of GAPDH and presented in the bar graphs

We first examined the effect of HC-1119 on the proliferation of Hs578T and SUM159PT TNBC cells, and found no obvious effect of HC-1119 treatment on the proliferation of these cells (Figure S3). Since DHT promotes cell metastasis via activating AR in breast cancer [26, 27], we next examined the effects of HC-1119 treatment on the migration and invasion of these cells. DHT treatment robustly increased the migration and invasion capabilities of both Hs578T and SUM159PT cell lines. HC-1119 treatment did not significantly alter the migration and invasion capabilities of these cells. However, HC-1119 treatment completely inhibited the DHT-stimulated migration and invasion capabilities of these cells (Fig. 4 A and B). Since cell migration and invasion abilities are positively correlated with epithelial-to-mesenchymal transition (EMT), we examined the effects of DHT and HC-1119 treatments on the expression levels of the epithelial marker cytokeratin 18 (K18) and mesenchymal markers fibronectin (FN) and vimentin. Of note, E-cadherin is not expressed in these TNBC cells. DHT treatment downregulated K18 protein expression but increased FN and vimentin protein expression, while HC-1119 treatment upregulated K18 expression but downregulated FN and vimentin expression (Fig. 4 C and D). These results demonstrate that HC-1119 can effectively restrict the migration and invasion capabilities of AR-positive TNBC cells stimulated by DHT through partial inhibition of the DHT-induced EMT.

### HC-1119 treatment inhibits the metastasis of AR-positive TNBC cells in mice

**Fig. 5:**
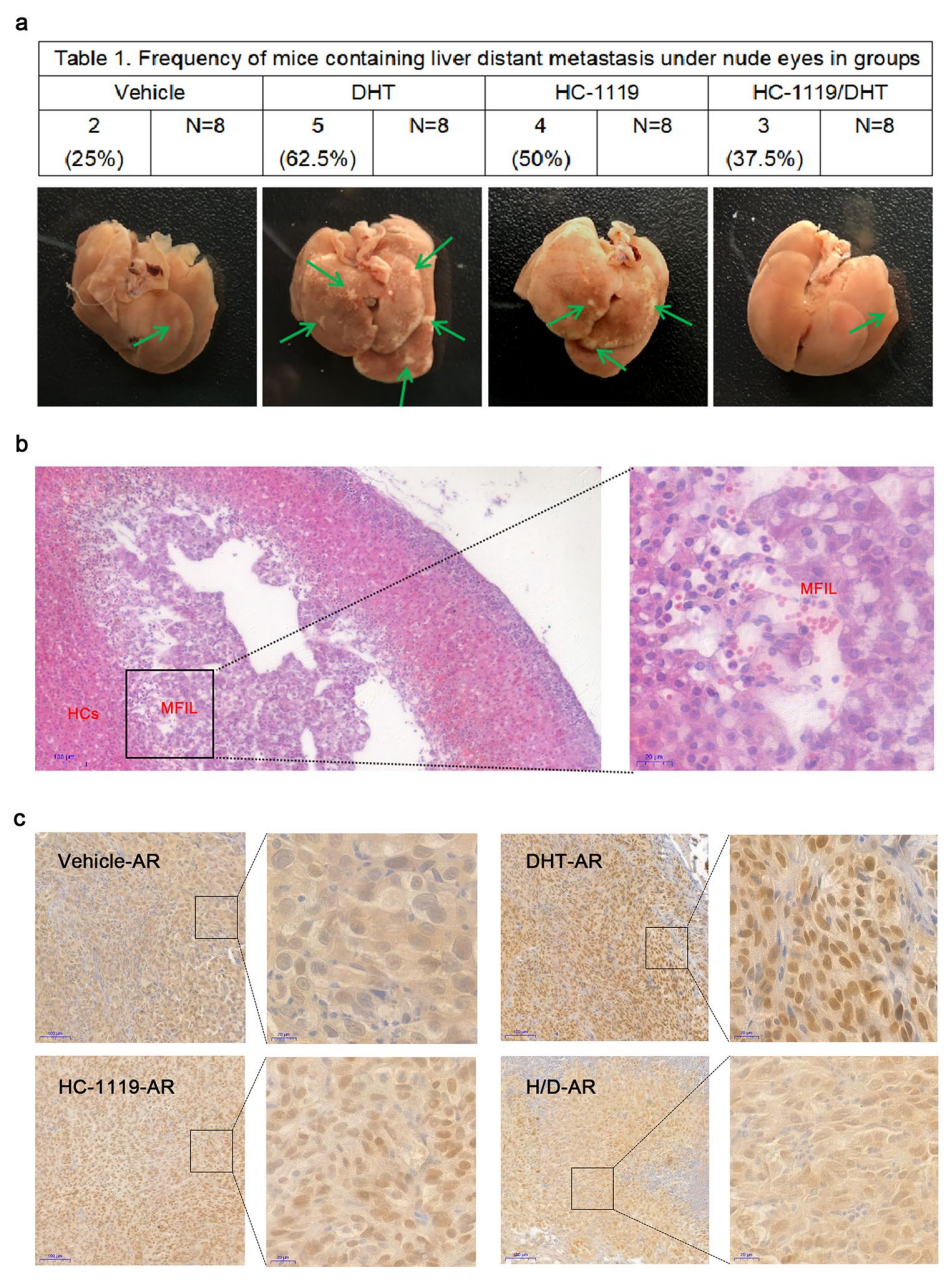
**HC-1119 treatment reduces DHT-promoted metastasis of AR-positive TNBC cells in mice (a)** Visible metastatic nodules in the liver. Two-million SUM159PT cells were injected into one of the fourth mammary gland fat pads of the 6-week-old BALB/c nude mice (n = 8). One week later, mice were treated with vehicle, DHT, HC-1119 or both DHT and HC-1119 as indicated for 31 days. The representative liver images with metastatic nodules (indicated by arrows) are presented. The number of mice with liver metastasis in each group is also presented in the table. **(b)** Representative H&E-stained liver sections with metastasis lesion. The boxed area in the left panel is enlarged in the right panel. MFIL, metastatic foci in liver. HCs, hepatic cords. **(c)** Immunohistochemical staining for AR (brown color). Tissue sections were prepared from xenograft tumors in the mammary gland fat pads of mice treated with vehicle, DHT, HC-1119 or both DHT and HC-1119 as indicated. Immunohistochemical staining for AR was carried out by using an AR antibody. The boxed areas are shown in higher magnification as indicated

We orthotopically inoculated SUM159PT cells into the mammary gland fat pads of female BALB/c nude mice to grow xenograft tumors, and treated these mice with vehicle, DHT, HC-1119 or both DHT and HC-1119 for 31 days. All of these treatments did not significantly affect the growth of the primary xenograft tumors derived from these cells (Figure S5). Two of the 8 vehicle-treated mice and 5 of the 8 DHT-treated mice developed liver metastasis, suggesting that DHT treatment promotes the metastasis of SUM159PT cells. However, only 3 of the 8 DHT and HC-1119-cotreated mice developed liver metastasis, and the number of metastatic nodules in these cotreated mice was also significantly reduced compared with that in the DHT-treated mice (Fig. 5 A and B). Immunohistochemistry staining for AR confirmed that DHT effectively induced AR to translocate into the nucleus, while HC-1119 treatment largely blocked DHT-induced nuclear localization of AR in the xenograft tumor cells (Fig. 5 C). These results suggest that HC-1119 treatment inhibits the androgen receptor signaling promoted liver metastasis of the SUM159PT AR-positive TNBC cells in vivo.

## Discussion

HC-1119 is a new second-generation AR antagonist that has been used in clinical trials to treat prostate cancer [29, 30]. Although lacking sufficient pharmacokinetics data, the effective concentrations of HC-1119 for inhibiting AR function are 0.5 µM in Hs578T cells and 10 µM in SUM159PT cells, which are much lower than the 4 to 50 µM of the regular Enzalutamide [31] and the 25 µM to 10 mM of bicalutamide used for TNBC cells [32–34]. The usage of lower dose may help to reduce side effects.

We found that HC-1119 treatment could inhibit DHT-promoted migration and invasion capabilities of the AR-positive TNBC cells, and this inhibition might be a consequence of reduced EMT plasticity of these TNBC cells. Previous studies have shown that DHT-activated AR interacts with demethylase LSD1 to repress E-cadherin transcription and upregulate vimentin transcription in the ER and AR positive MCF7 breast cancer cells, leading to EMT that increases cell migration and invasion [27]. Furthermore, AR activation can enhance the viability of cancer stem-like cells (CSCs) through an androgen paracrine action, and also facilitates the steaminess features of AR-positive cancer cells [19]. Our results indicate that HC-1119 could be an effective AR inhibitor to suppress androgen-promoted migration and invasion of AR-positive TNBCs.

HC-1119 at a dose of 7 mg/kg given to mice every 2 days could reduce DHT-promoted metastasis of TNBC cells in vivo. Early studies showed that testosterone levels are fluctuating, and plasma concentration of DHT is maintained in a dynamic balance during menstrual cycle, which is higher in premenopause women and lower in postmenopause women [35, 36]. Hence, it should be meaningful to examine plasma concentration of DHT (PC-DHT) routinely in TNBC patients. Furthermore, since the DHT-induced nuclear translocation of AR is a key step for AR signaling to promote metastasis of TNBC [8, 9], the ratio of nuclear AR to cytoplasmic AR, together with the PC-DHT, may be used to assess whether a patient with AR-positive TNBC is sensitive to HC-1119 treatment and can receive benefits from HC-1119 treatment.

It is known that DHT binds and activates AR, which in turn increases AR protein through multiple pathways [37–40]. Our results suggest that HC-1119 can completely block the increase in AR protein induced by DHT, indicating that HC-1119 can effectively suppress AR function in TNBC cells. In addition, our assays suggest that either DHT or HC-1119 treatment did not significantly alter the proliferation or apoptosis of TNBC cells (data not shown), suggesting that AR function is not essential for the growth or survival of TNBC cells. A previous study also demonstrated that DHT treatment of the patient-derived xenografts developed from patients with AR-positive TNBCs mainly inhibits EMT, angiogenesis, and WNT/β-catenin signaling [41].

In this study, we have evaluated the efficacy of HC-1119 in inhibition of DHT-activated AR function in AR-positive TNBC cells. However, we have not compared the efficacy of HC-1119 with the regular enzalutamide in these cells, which will be planned in our future experiments. Furthermore, we only studied the response of DHT-treated TNBC cells, but accumulating evidence suggests that AR also plays an important role in ER-positive breast cancer cells [20, 38, 42, 43]. Thus, it will be interesting to test the inhibitory effects of HC-1119 on ER-positive breast cancer cells. Moreover, the crosstalk between AR signaling and other pathways such as PI3K/AKT/mTOR, WNT/β-catenin and MAPK has been shown being involved in the abnormal activation of AR and the resistance to AR antagonist [37, 44, 45]. It would be important to test whether combinatorial treatment with HC-1119 and other pathway inhibitors can achieve a better result. Finally, the pharmacokinetics, safety and tolerability of HC-1119 should be further studied.

In summary, this study demonstrated that HC-1119 can effectively inhibit DHT-increased AR protein and DHT binding-induced AR-nuclear translocation, and thus block DHT-promoted migration, invasion and metastasis of TNBC cells. These results suggest that HC-1119 is a new second-generation AR inhibitor that might be used to treat AR-positive TNBCs. This is the first study showing that HC-1119 may be used to treat other types of AR-positive cancers such as AR-positive TNBC in addition to the prostate cancer.

## Electronic supplementary material

Supplementary material 1

## Data Availability

The original data of this study are available from the corresponding authors upon request.
