# Supplementary material for: HC-1119, a deuterated Enzalutamide, inhibits Migration, Invasion and Metastasis of the AR-positive triple-negative breast Cancer cells
Source: Mol Biol Rep. 2022 Aug 12;49(10):9231–40. doi: 10.1007/s11033-022-07749-8 (PMC9515013; doi:10.1007/s11033-022-07749-8)
Supplement: Supplementary file 1 — Supplementary material 1 [file 11033_2022_7749_MOESM1_ESM.docx]

**SUPPLEMENTARY FIGURE**

**
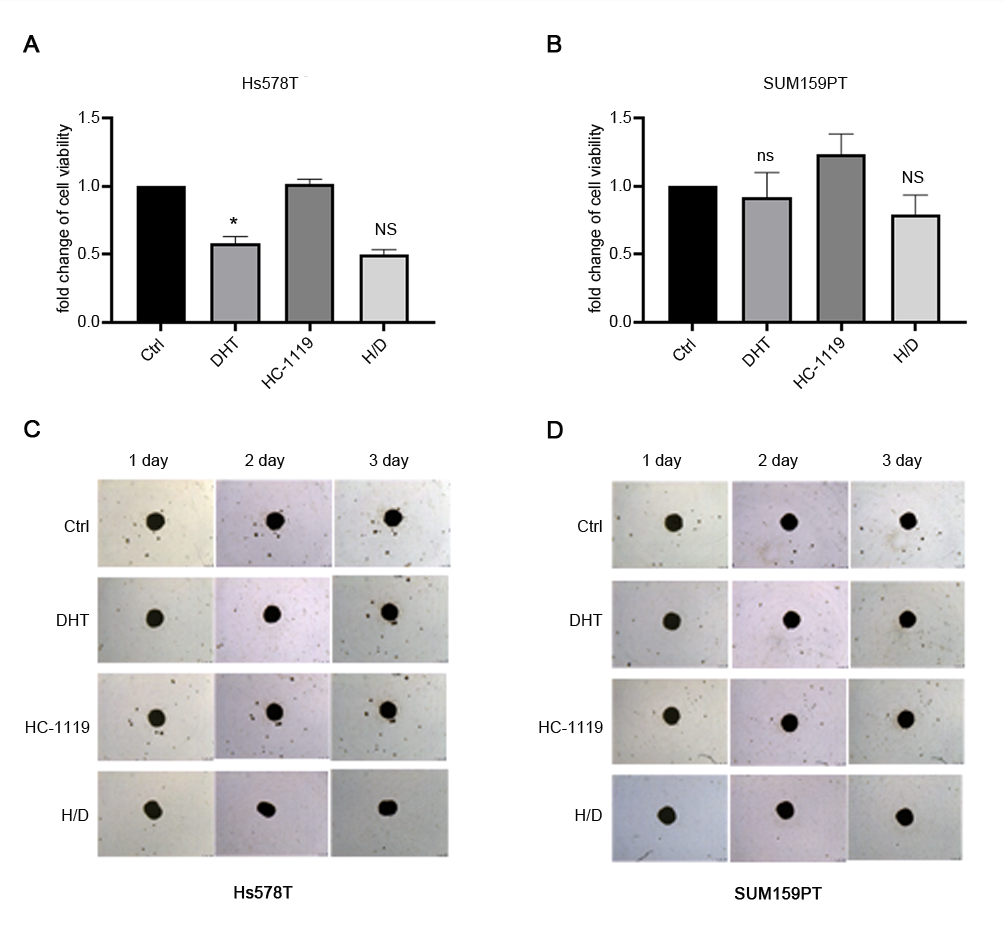
 Figure S3. DHT has little influence in cell growth of TNBC cells in vitro.**

**(A, B)** cell viability of Hs578T and SUM259PT under the treatment of DHT and HC-1119. Cells were treated as described in supplementary method. Relative cell number was quantified by CCK-8 kit. * represents p<0.05 (relative to Ctrl group), “ns”, p＞0.05 (relative to Ctrl group), “NS”, p＞0.05 (relative to DHT group) by Student’s t test. **(C, D)** cell growth ability of Hs578T and SUM259PT under the treatment of DHT and HC-1119. Images shows sphere size when treated for 1 day, 2 days and 3 days. Experiments above were repeated at least three times and the representative data were presented.

**METHOD**

**Cell growth assay**

Hs578T and SUM159PT were seeded in 96-well plates at 3×10^3^ cells/well, cultured overnight, and treated with 200 μl 10% CSS medium containing 5 nM DHT and HC-1119, Hs578T with 0.5 μM and SUM159PT with 10 μM, respectively or in combination, equal volume of DMSO served as control for 5 days. Cell Counting Kit-8 (CCK-8) assay were exploited to measuring cell viability. Briefly, plates added with 20 μl of CCK-8 in each well were incubated at 37 ℃ for 4 hours. The absorbance was measured at 450 nm by using a SpectraMax i3 spectrophotometer (Molecular Devices, Sunnyvale, CA, USA).


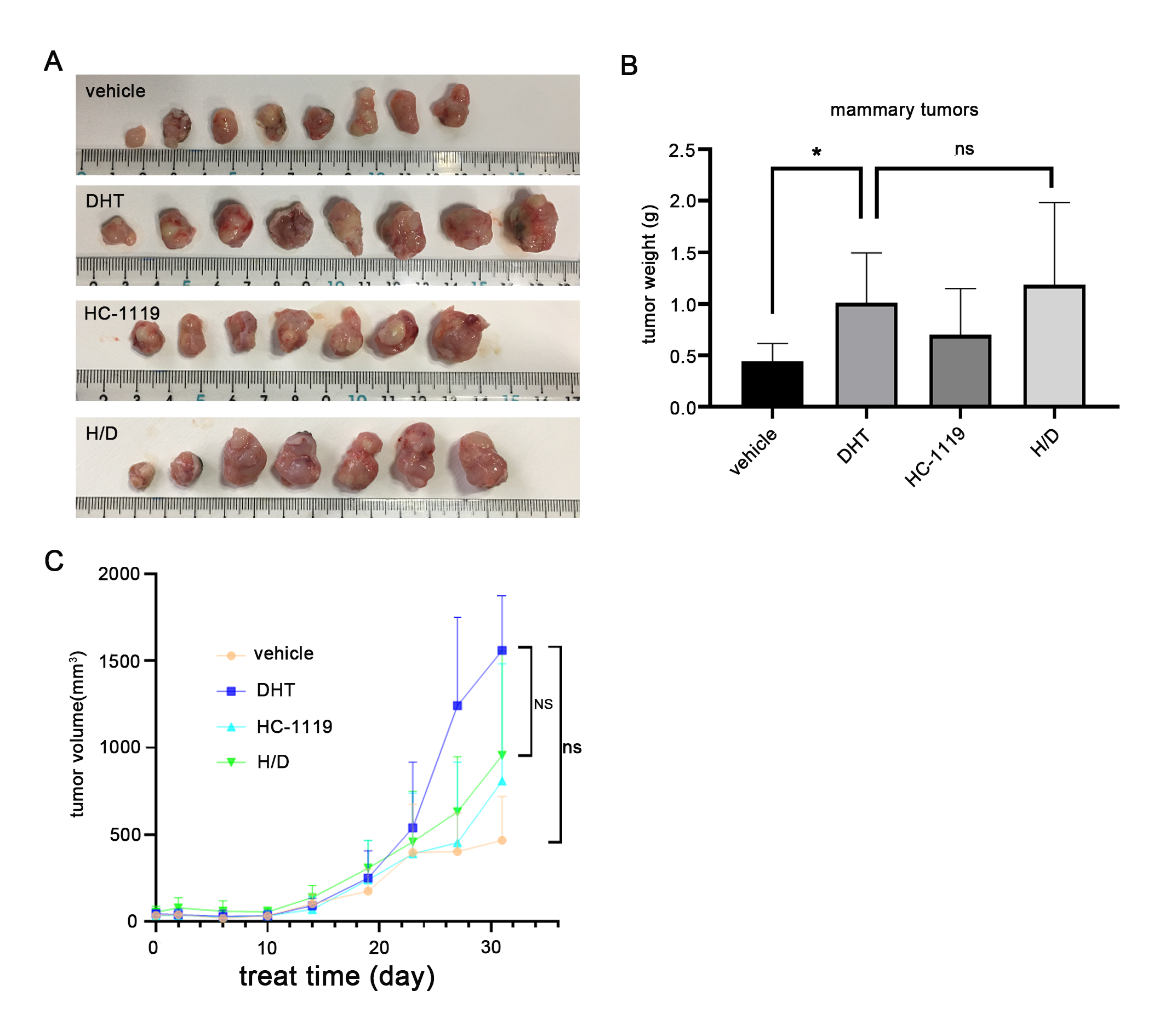


**Figure S5. The impact of HC-1119 on tumor growth in vivo.**

**(A)** Two million SUM159PT cells were injected into one of the fourth pair mammary gland fat pads of nude mice. Image of individual tumors derived from xenograft model treated with vehicle or DHT or HC-1119 as indicated for 31 days. **(B)** Average weights of wet tumors collected from mice shown in Panel A. The number of tumors weighed in each group is indicated. *, p < 0.05 by Student’s t test. **(C)** Tumor growth curves. Tumor volume was measured as described in the Materials and Methods section and presented as Mean. “ns”, p＞0.05 relative to Vehicle, “NS”, p＞0.05 relative to DHT by Student’s t test.

**METHOD**

**Mammosphere growth assay**

Every 3×10^3^ cells of Hs578T and SUM159PT in 100 μl 10% CSS medium were added to wells of ultra-low attachment U bottom 96-well plates (Corning). Mammospheres were grown after 24 hours’ culturing. Cells were treated with 5 nM DHT or HC-1119 (Hs578T with 0.5 μM, SUM159PT with 10 μM) or in combined, or equal volume of DMSO served as control for 3 days, with 8 parallel samples for each group. Each sphere was imaged after treated for 1 day, 2 days and 3 days.
